# Atrial Natriuretic Peptide Promotes Neurite Outgrowth and Survival of Cochlear Spiral Ganglion Neurons *in vitro* Through NPR-A/cGMP/PKG Signaling

**DOI:** 10.3389/fcell.2021.681421

**Published:** 2021-06-23

**Authors:** Fei Sun, Ke Zhou, Ke-yong Tian, Xin-yu Zhang, Wei Liu, Jie Wang, Cui-ping Zhong, Jian-hua Qiu, Ding-jun Zha

**Affiliations:** ^1^Department of Otolaryngology-Head and Neck Surgery, Xijing Hospital, Fourth Military Medical University, Xi’an, China; ^2^Department of Laboratory Medicine, Institute of Clinical Laboratory Medicine of PLA, Xijing Hospital, Fourth Military Medical University, Xi’an, China; ^3^Department of Otolaryngology-Head and Neck Surgery, The Affiliated Children Hospital of Xi’an Jiaotong University, Xi’an, China; ^4^Department of Otolaryngology-Head and Neck Surgery, The 940th Hospital of Joint Logistics Support Force of PLA, Lanzhou, China

**Keywords:** sensorineural hearing loss, spiral ganglion neuron, atrial natriuretic peptide, natriuretic peptide receptors, cGMP, neurite outgrowth, neuroprotection

## Abstract

Sensorineural hearing loss (SNHL) is a dominant public health issue affecting millions of people around the globe, which is correlated with the irreversible deterioration of the hair cells and spiral ganglion neurons (SGNs) within the cochlea. Strategies using bioactive molecules that regulate neurite regeneration and neuronal survival to reestablish connections between auditory epithelium or implanted electrodes and SGN neurites would become attractive therapeutic candidates for SNHL. As an intracellular second messenger, cyclic guanosine-3’,5’-monophosphate (cGMP) can be synthesized through activation of particulate guanylate cyclase-coupled natriuretic peptide receptors (NPRs) by natriuretic peptides, which in turn modulates multiple aspects of neuronal functions including neuronal development and neuronal survival. As a cardiac-derived hormone, atrial natriuretic peptide (ANP), and its specific receptors (NPR-A and NPR-C) are broadly expressed in the nervous system where they might be involved in the maintenance of diverse neural functions. Despite former literatures and our reports indicating the existence of ANP and its receptors within the inner ear, particularly in the spiral ganglion, their potential regulatory mechanisms underlying functional properties of auditory neurons are still incompletely understood. Our recently published investigation revealed that ANP could promote the neurite outgrowth of SGNs by activating NPR-A/cGMP/PKG cascade in a dose-dependent manner. In the present research, the influence of ANP and its receptor-mediated downstream signaling pathways on neurite outgrowth, neurite attraction, and neuronal survival of SGNs *in vitro* was evaluated by employing cultures of organotypic explant and dissociated neuron from postnatal rats. Our data indicated that ANP could support and attract neurite outgrowth of SGNs and possess a high capacity to improve neuronal survival of SGNs against glutamate-induced excitotoxicity by triggering the NPR-A/cGMP/PKG pathway. The neuroregenerative and neuroprotective effects of ANP/NPRA/cGMP/PKG-dependent signaling on SGNs would represent an attractive therapeutic candidate for hearing impairment.

## Introduction

Hearing loss has become a global health and economic problem affecting approximately 20% of the world’s population (one in five people) suffering from different degrees of sensory disorder, while more than 430 million people experience disabling hearing loss, according to the first World Report on Hearing recently announced by the World Health Organization^1^. As a major type of hearing loss, sensorineural hearing loss (SNHL) can be caused by various insults such as acoustic trauma, ototoxic drugs, infections, aging, genetic mutations, and tumor (He et al., 2017; McLean et al., 2017; Zhu et al., 2018; Fang et al., 2019; Jiang et al., 2020; Qian et al., 2020; Lv et al., 2021), resulting in irreversible deterioration of sensory cells, e.g., the cochlear hair cells (HCs) (He et al., 2016; Liu et al., 2016; Li A. et al., 2018; Xia et al., 2019; Zhang S. et al., 2019; Zhang et al., 2020c) and auditory neurons (Guo et al., 2016; Yan et al., 2018; Liu W. et al., 2019; Guo et al., 2020, 2021). The cochlear HCs in the organ of Corti are mechanosensory cells which convert mechanical sound waves into primary acoustic signals (Wang et al., 2017; Zhu et al., 2018; Liu Y. et al., 2019; Qi et al., 2019, 2020). The spiral ganglion neurons (SGNs) are the cochlear primary afferent neurons which perform a crucial function in hearing by conducting auditory information from the sensory HCs to the auditory center within the brain (Nayagam et al., 2011). Because the regenerative capacity of HCs and SGNs in the mature mammalian cochlea is very limited (Cox et al., 2014; Lu et al., 2017; Cheng et al., 2019; Tan et al., 2019; Zhang et al., 2020a,b), irreversible death of cochlear HCs followed by a progressive degeneration of SGNs ultimately causes permanent hearing loss (He et al., 2019; Ding et al., 2020; He et al., 2020; Roccio et al., 2020; Zhou et al., 2020).

Over a longer period, various strategies have been developed in an attempt to treat or at least prevent further progression of SNHL. Unfortunately, the therapeutic efficacy for patients with hearing impairment is still unsatisfactory. Novel therapeutics based on genetic and cell transplantation techniques appear to be a promising approach to restoring hearing function, including substitution or regeneration of HCs and/or SGNs by means of stem and progenitor cells transplantation (Li D. et al., 2019; Li G. et al., 2019; Tang et al., 2019; Zhao et al., 2019; Fang et al., 2020; Xia et al., 2020; Yang et al., 2020), as well as *in situ* reprogramming of adjacent supporting cells or glial cells into functional HCs or neurons (Chen et al., 2017; Cheng et al., 2017; McLean et al., 2017; Zhang et al., 2017; Noda et al., 2018; You et al., 2018; Zhang et al., 2018; Zhang Y. et al., 2019; Li et al., 2020; Waqas et al., 2020; Zhang et al., 2020c). Moreover, transplantation of stem/progenitor cells into the human cochlea is technically demanding and is accompanied by the possible risks of developing tumors (Nishimura et al., 2012). Currently, auditory prosthesis such as cochlear implants (CIs) are a favorable solution for patients with profound SNHL, which can substitute for the missing HCs to directly stimulate the surviving neurons from the auditory nerve (Ma et al., 2019). The effectiveness of such neural prosthetic devices relies not only on the integrated interface where the implant’s electrodes make contact with peripheral neurites of SGNs but also on the number of functional auditory neurons being stimulated. In order to optimize the outcome of cochlear implantation, some biological approaches are attempted to preserve the residual auditory neurons from degeneration after the loss of auditory HCs or to promote regeneration and outgrowth of neurites from SGNs toward the electrode array (Kwiatkowska et al., 2016; Li et al., 2017; Guo et al., 2019). Consequently, new strategies using bioactive molecules that facilitate neurite regeneration and neuronal survival to reestablish the synaptic connections between the auditory sensory epithelium or implanted electrode array and SGNs neurites would become attractive therapeutic candidates for hearing impairment.

Cyclic guanosine-3’,5’-monophosphate (cGMP) acts as an important secondary messenger which mediates various biological functions through three effector molecules comprising cGMP-dependent protein kinases (PKG, also known as cGK), cGMP-gated ion channels, and cGMP-regulated isoforms of phosphodiesterases. The pathway involving cGMP may offer a unique signaling mechanism in modulating neuronal development and synaptic plasticity associated with neurite outgrowth and pathfinding, neuronal survival, neuronal excitability, and the sensory transduction cascades associated with vision and olfaction (Zhao and Ma, 2009; Zhao et al., 2009). Under the physiology condition, cGMP can be catalyzed by two categories of molecularly distinct guanylate cyclases (GCs), including soluble GC which recognizes its ligand nitric oxide and particulate GC-coupled receptors that are specifically activated by natriuretic peptides (NPs). Specifically, the transmembrane GC-coupled natriuretic peptide receptors (NPRs), NPR-A and NPR-B, interact with three NPs, in turn leading to the activation of the intracellular GC domain and synthesis of cGMP. Owing to the absence of GC activity, the clearance receptor NPR-C can remove circulating NPs through ligand binding, internalization, and degradation (Potter et al., 2009).

Atrial natriuretic peptide (ANP) is a cardiac-derived hormone predominantly synthesized and secreted by the cardiac atria, which dynamically regulates blood pressure through decreasing water and sodium loads in the circulatory system. Through binding to its specific receptors on the cell surface, NPR-A (also known as NPR1 or GC-A) and NPR-C, ANP exerts a fundamental role in the modulation of cardiovascular homeostasis (Potter et al., 2009). ANP, brain NP (BNP), and C-type NP (CNP) are structurally related peptides belonging to members of the NP family that are widely distributed in the mammalian central nervous system (CNS) (Cao and Yang, 2008; Mahinrad et al., 2016), especially in peripheral sensory organs such as the dorsal root, trigeminal, retinal, and cochlear ganglia (Lamprecht and Meyer zum Gottesberge, 1988; Furuta et al., 1995; Abdelalim and Tooyama, 2010; Xu et al., 2010; Loo et al., 2012; Abdelalim et al., 2013; Vilotti et al., 2013; Fitzakerley and Trachte, 2018). Recent research has shown the involvement of the BNP/NPR-A signaling pathway in the modulation of nociceptive processing for pain (Zhang et al., 2010) and itch responses (Solinski et al., 2019). Besides, cGMP signaling elicited by the CNP/NPR-B pathway regulates neurite outgrowth and pathfinding of sensory ganglion neurons within DRG (Schmidt et al., 2002, 2007; Kishimoto et al., 2008; Schmidt et al., 2009; Zhao et al., 2009; Xia et al., 2013; Ter-Avetisyan et al., 2014; Schmidt et al., 2016; Dumoulin et al., 2018a,b; Schmidt et al., 2018; Ter-Avetisyan et al., 2018; Tröster et al., 2018) and cochlear spiral ganglion (SG) (Lu et al., 2011, 2014; Ter-Avetisyan et al., 2014, 2018; Wolter et al., 2018; Schmidt and Fritzsch, 2019) during development. Additionally, all three NPs possess neuroprotective ability on retinal and central neurons through GC-coupled NPR stimulation (Fiscus et al., 2001; Cheng Chew et al., 2003; Kuribayashi et al., 2006; Ma et al., 2010; Colini Baldeschi et al., 2018). All these clues indicate that NPs and their receptors could probably be associated with the maintenance of various aspects of neuronal functions.

The existence of ANP and receptors in the sensory and secretory compartments of the mammalian inner ear has been systematically elucidated in a great number of literatures (Lamprecht and Meyer zum Gottesberge, 1988; Meyer and Lamprecht, 1989; Gottesberge et al., 1991; Koch et al., 1992; Yoon and Hellström, 1992; Yoon and Anniko, 1994; Furuta et al., 1995; Meyer Zum Gottesberge et al., 1995; Krause et al., 1997; Suzuki et al., 1998; Seebacher et al., 1999; Yoon et al., 2012; Sun et al., 2013, 2014; Yoon et al., 2015; Sun et al., 2020), while recent works also show that ANP receptors are distributed in rat cochlear SG and modiolus of the guinea pig (Lamprecht and Meyer zum Gottesberge, 1988; Furuta et al., 1995). However, the distribution patterns and potential functions of ANP and its receptors in the neural elements of the inner ear remain unclear. In our previous studies, the expression patterns of ANP and its receptors were investigated in the cochlear SG, which provided direct evidence for the presence and synthesis of ANP and its receptors in the neural region of the cochlea (Sun et al., 2013, 2014). We recently demonstrated that ANP might promote neurite outgrowth of cochlear SGNs by triggering the NPR-A/cGMP/PKG pathway in a dose-dependent manner (Sun et al., 2020). In the current study, the influence of ANP on neurite outgrowth, neurite attraction, and neuronal survival of rat SGNs was evaluated by employing organotypic explant and dissociated neuron cultures *in vitro*. All these findings demonstrated that ANP may promote neurite outgrowth and neuronal survival of SGNs by activating the NPR-A/cGMP/PKG pathway, not via interaction with the NPR-C pathway. ANP may perform a vital role in the normal neuritogenesis of cochlear auditory neurons during development of the inner ear and may enhance neurite regeneration and neuronal viability of SGNs, thus representing a potential therapeutic candidate for hearing impairment.

## Materials and Methods

### Animals and Tissue Preparation

**FIGURE 1:**
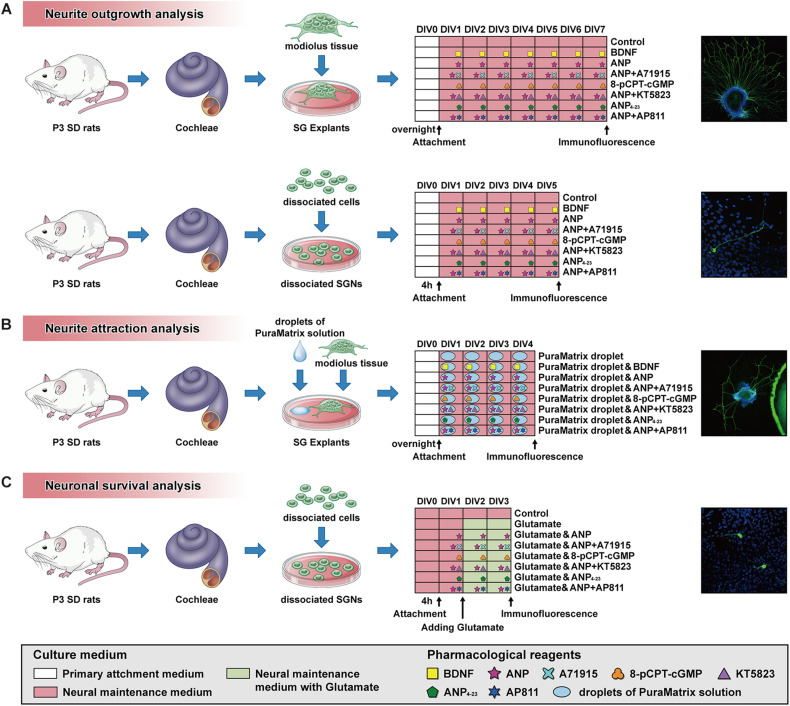
Schematic summary of the protocol and timeline for SG explant or dissociated SGN cultures used for analysis of neurite outgrowth **(A)**, neurite attraction **(B)** and neuronal survival **(C)**.

All experimental procedures have been permitted by the Animal Care Committee of the Fourth Military Medical University, China. Animals used for the study were provided by the Laboratory Animal Center of the Fourth Military Medical University. Sprague-Dawley rats of postnatal day 3 (P3) and P7 were used for analyses. For the preparation of cochlear sections and cultures, the rat pups were sacrificed by rapid decapitation, and their skulls were opened midsagitally. All cochleae were promptly separated from the temporal bone under a dissecting microscope, rinsed with ice-cold Hank’s Balanced Salt Solution (HBSS; Thermo Fisher Scientific), and collected for further use. A schematic summary of the protocol and timeline for SG explant or dissociated SGN cultures used in this study is illustrated in Figure 1.

### Immunofluorescent Analysis of ANP and Its Receptors on Cochlear Sections

For cochlear cryosections, cochleae extracted from P7 rats were superfused with fresh 4% paraformaldehyde solution through the round and oval windows and further immersed in an identical fixative solution overnight at 4°C. The cochlear tissues were decalcified for 2 days in 5% EDTA followed by dehydration in 30% sucrose overnight at 4°C. After being embedded in Tissue-Tek OCT compound (Sakura Finetek) at −20°C, tissues were oriented to obtain mid-modiolar cross sections and then cut on a cryostat microtome into 12-μm-thick sections. Cryosections were then placed onto poly-L-lysine-coated slides and allowed to dry overnight at room temperature (RT) before performance of immunofluorescence staining.

For immunohistochemistry, the cochlear sections were rinsed with phosphate-buffered saline (PBS) and incubated in a blocking and permeabilizing solution composed of 5% bovine serum albumin (BSA; Sigma-Aldrich) and 0.1% Triton X-100 dissolved in PBS for 40 min at 37°C. All slides were immersed into the antibody solution (1% BSA and 0.1% Triton X-100 in PBS) supplemented with the following primary antibodies at a concentration of 1:500 at 4°C overnight: monoclonal mouse anti-β-III tubulin (TUJ1) antibody (Abcam, #ab78078), polyclonal rabbit anti-ANP antibody (Thermo Fisher Scientific, #PA5-29559), polyclonal rabbit anti-NPR-A antibody (Thermo Fisher Scientific, #PA5-29049), and polyclonal rabbit anti-NPR-C antibody (Thermo Fisher Scientific, #PA5-96947). Immunolabeling was further visualized with Alexa Fluor 488-conjugated donkey anti-mouse IgG (1:500; Thermo Fisher Scientific, #A-21202) and Alexa Fluor 594-conjugated donkey anti-rabbit IgG (1:500; Thermo Fisher Scientific, #A-21207) diluted in an antibody solution at a concentration of 1:500 for 2 h at RT. Cochlear specimens randomly processed for staining in parallel without incubation with primary antibodies were served as a negative control. After counterstaining with Hoechst 33342 at 1:1,000 (Thermo Fisher Scientific) for 15 min at RT, all samples were subsequently mounted with the Prolong Gold anti-fade mounting medium (Thermo Fisher Scientific). All confocal images acquired under a confocal microscope system (FV1000; Olympus) were converted to TIFF format using a confocal software (FV10-ASW 4.2; Olympus) and processed for optimal brightness and contrast with the Adobe Photoshop software (CC 2020; Adobe Systems).

### Analysis of Neurite Outgrowth in SG Explant Cultures

The experimental protocol for cochlear SG explant culture was performed according to a previous study (Sun et al., 2016) with a minor modification. Briefly, the membranous labyrinth was revealed after removal of the cochlear capsule from P3 rat cochleae under the dissecting microscope. After the spiral ligament, stria vascularis, and basilar membrane were separated from the modiolus, the remaining spiral lamina containing SG was cautiously dissected into equal pieces of approximately 500 μm. Subsequently, each dissected tissue was adhered to a single 15-mm glass-bottom culture dish (Advance BioScience), previously coated with Cell-Tak (BD Biosciences, #354240), and filled with 100 μl of primary attachment medium consisting of Dulbecco’s modified Eagle medium (DMEM), 10% fetal bovine serum (FBS), 25 mM HEPES, and 1% penicillin–streptomycin (all Thermo Fisher Scientific). Samples were kept overnight in an atmosphere of 5% CO_2_ and 95% humidity at 37°C. After attachment of explants, the culture medium was aspirated, and 100 μl of 20% Matrigel (BD Biosciences, #356234) mixture diluted with the attachment medium was directly dropped onto each explant culture. Immediately, the culture dish was transferred to the incubator to initiate gelation of the Matrigel for 10 min.

For neurite outgrowth assay, SG explants were cultured in neural maintenance medium consisting of DMEM/Ham’s F12 medium (DMEM/F12) supplemented with 1x B27 and 1x N2 (all Thermo Fisher Scientific) and 1% penicillin–streptomycin and simultaneously treated with distinct pharmacological reagents. Explants incubated in medium supplemented with or without 20 ng/ml recombinant brain-derived neurotrophic factor (BDNF; PeproTech, #450-02) were served as control cultures. Cultures from other experimental groups were maintained in medium supplemented with 1 μM of ANP (Caymanchem, #24276), 1 μM of ANP plus 1 μM of NPR-A antagonist A71915 (Bachem, #4030385), 1 μM of the cell-permeable analog of cGMP 8-(4-chlorophenylthio) guanosine-3’,5’-cyclic monophosphate (8-pCPT-cGMP; Sigma-Aldrich, #C5438), 1 μM of ANP plus 1 μM of the PKG inhibitor KT5823 (Sigma-Aldrich, #K1388), 1 μM of the specific NPR-C agonist ANP_4–23_ (Echelon Biosciences, #155-40), or 1 μM of ANP plus 1 μM of the NPR-C antagonist AP811 (Tocris Bioscience, #5498). In each experimental group, five explants were incubated at 37°C with 5% CO_2_ for 7 days prior to fixation. The medium was changed every other day for the duration of the experiment.

### Analysis of Neurite Outgrowth in Dissociated SGN Cultures

Dissociated SGN cultures were conducted in accordance with the procedures from a previous document (Tuft et al., 2013). The cochlear ducts of P3 rats containing the organ of Corti, spiral ligament, and stria vascularis were sequentially dissected away to collect modiolus tissues harboring the SGNs. The tissues were then applied to enzymatic dissociation by using 0.1% collagenase type IV and 0.25% trypsin in Ca^2+^/Mg^2+^-free HBSS (all Thermo Fisher Scientific) at 37°C for 20 min. After enzymatic digestion was inactivated by adding equal volumes of 10% FBS, the tissues were mechanically triturated into cell suspensions through a fire-polished glass Pasteur pipette. The dissociated SG cells were resuspended in neural maintenance medium and seeded at a density of 2.0 × 10^5^ cells/poly-L-lysine-coated (Thermo Fisher Scientific) dish for 4 h.

For neurite outgrowth assay, the attached SGNs were maintained in culture medium supplemented with or without the following reagents consistent with those in SG explant cultures: 20 ng/ml BDNF, 1 μM ANP, 1 μM ANP plus 1 μM A71915, 1 μM 8-pCPT-cGMP, 1 μM ANP plus 1 μM KT5823, 1 μM ANP_4–23_, or 1 μM ANP plus 1 μM AP811. In each group, five culture dishes containing dissociated cells were replenished with a fresh medium every other day and transferred to a 5% CO_2_ incubator at 37°C for 5 days before fixation.

### Analysis of Neurite Attraction in SG Explant Cultures

The procedures for evaluating the neurite attraction of SG explants cultures were adopted from procedures described previously with several modifications (Frick et al., 2017). Briefly, 1% PuraMatrix solution (BD Biosciences, #354250) was diluted to 0.25% solution with sterile ultrapure water (Milli-Q, Merck) alone or diluted with ultrapure water containing the following reagents to obtain a 0.25% PuraMatrix mixture: 20 ng/ml BDNF, 1 μM ANP, 1 μM ANP plus 1 μM A71915, 1 μM 8-pCPT-cGMP, 1 μM ANP plus 1 μM KT5823, 1 μM ANP_4–23_, or 1 μM ANP plus 1 μM AP811. Each resulting PuraMatrix hydrogel (diluted solution or mixture) was briefly sonicated for 30 min for homogenization. Subsequently, 5-μl droplets of 0.25% PuraMatrix hydrogel were transferred into Cell-Tak-coated culture dishes, and dishes were maintained by incubation at 37°C for 30 min before settling of SG explants. Each dissected SG explant was plated at approximately 0.5 mm next to a hydrogel droplet in a culture dish and settled to attach as described above. After attachment, all explants were cultured in the neural maintenance medium for 4 days prior to fixation, and the culture medium was refreshed every other day. For each condition, five explants were maintained for neurite attraction study.

### Analysis of Neuronal Survival in Dissociated SGN Cultures With Glutamate Excitotoxicity

The dissociated cells from modiolus tissues obtained as described above were incubated in neural maintenance medium at the same density of 2.0 × 10^5^ cells per dish. On the first day *in vitro* (DIV1), dissociated cells from different experimental groups were maintained in a medium supplemented with or without the reagents as follows: 1 μM ANP, 1 μM ANP plus 1 μM A71915, 1 μM 8-pCPT-cGMP, 1 μM ANP plus 1 μM KT5823, 1 μM ANP_4–23_, or 1 μM ANP plus 1 μM AP811. At DIV2, 100 μM of L-glutamic acid (glutamate, Glu; Sigma-Aldrich, #G5889) was added to the culture medium of different experimental cultures to induce excitotoxicity, and all cultures were maintained for two additional days before fixation. Concurrently, cells incubated in the neural maintenance medium alone were served as a baseline control. In each group, five culture dishes seeded with dissociated SGNs were maintained for neuronal survival study.

### Immunofluorescence and Statistical Analysis

Following the culture period, all SG explants and dissociated SGNs were firstly fixed with paraformaldehyde for immunofluorescent analysis. All specimens were then permeabilized and blocked with a PBS solution containing 5% BSA and 0.1% Triton X-100, followed by incubation with the mouse primary antibody against TUJ1 (diluted 1:500) and Alexa Fluor 488-conjugated donkey anti-mouse IgG (diluted 1:500) to visualize the neural components. After nuclear counterstaining with Hoechst 33342 (diluted 1:1,000), all samples in the culture dishes were mounted with the Prolong Gold medium. Images of explant and cell cultures were photographed on the confocal microscope and analyzed for neurite tracing by using the “Neurite Tracer” function in the ImageJ software (version 1.46r; National Institute of Health) according to a previous study (Kempfle et al., 2018). The number and lengths of neurite outgrowth from the SG explants, together with the neuronal number and neurite length of dissociated SGNs, were measured and analyzed. Besides, the capacity of the PuraMatrix hydrogel to attract neurites was evaluated according to the previous research (Frick et al., 2017), by quantifying the probability of neurite attachment to hydrogel surfaces (estimated by the ratio of the number of explants with extended neurites that attached to hydrogel surfaces to the number of explants with extended neurites that contacted hydrogel droplets).

Furthermore, all experimental data are shown as the means ± standard error of the mean (means ± SEM) and analyzed by using the Statistical Program for Social Science software (SPSS, version 22.0; IBM Inc.). One-way analysis of variance (ANOVA) followed by Bonferroni’s *post hoc* test was used to assess the statistical significance. *P*-values less than 0.05 (*P* < 0.05) was accepted as statistically significant.

## Results

### Expression of ANP and Its Receptors in Cochlear SGNs of Postnatal Rats

**FIGURE 2:**
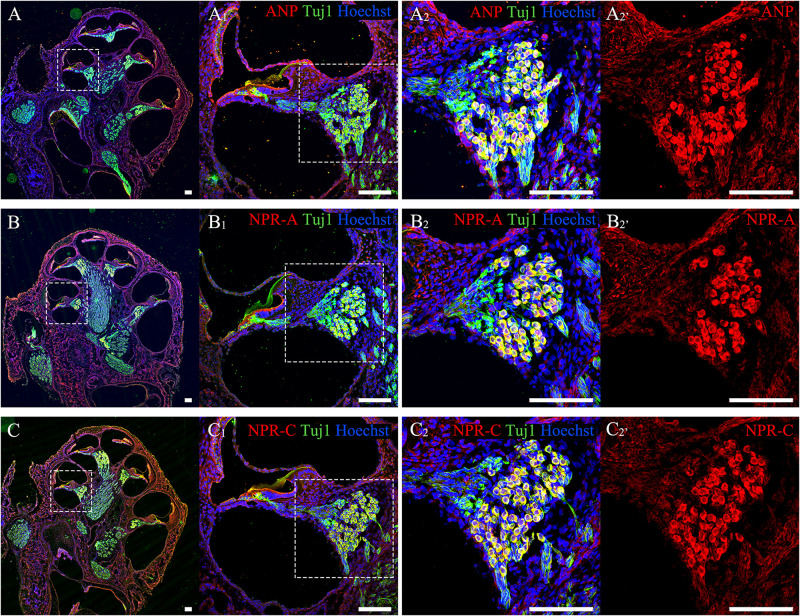
Immunolocalization of ANP, NPR-A and NPR-C in SGNs within cochlear SG from rats at P7. **(A–C)** Gross view of cryosectioned cochlear tissues triple-labeled with antibodies against neural marker β-III Tubulin (TUJ1, green), ANP/NPR-A/NPR-C (red) and Hoechst (blue). **(A_1_,B_1_,C_1_)** High-magnification views of the boxed region of **(A–C)**. **(A_2_,B_2_,C_2_)** Higher-magnification images of the boxed region of **(A_1_,B_1_,C_1_)** reveal that ANP/NPR-A/NPR-C is colocalized with TUJ1-positive SGNs, respectively. **(A_2’_,B_2’_,C_2’_)** Immunoreactivities of ANP, NPR-A, and NPR-C are present in the SGNs. Scale bars = 100 μm.

To confirm the distribution patterns of ANP and its receptors within the cochlear SG of postnatal rats in our previous publications, anti-ANP, NPR-A, and NPR-C immunolabeling were performed on cochlear cryosections from P7 rats co-immunostained with antibodies against neuron-specific β-III tubulin. As shown in Figure 2, ANP (Figure 2A) and its receptors’ (Figures 2B,C) immunoreactivities were observed in the SG regions along the length of the cochlear tonotopic axis without any noticeable apical-to-basal gradients. The representative images of cryosectioned tissues from the mid-cochlear turn revealed that the immunoreactivities of ANP (Figure 2A_2_), NPR-A (Figure 2B_2_), or NPR-C (Figure 2C_2_) were colocalized with TUJ1-positive neuronal somata of SGNs, which was consistent with our previous results (Sun et al., 2013, 2014, 2020). The similar distribution patterns of ANP (Figure 2A_2’_), NPR-A (Figure 2B_2’_), and NPR-C (Figure 2C_2’_) were observed in the cochlear SGNs, and they were predominantly immunoreactive in the neuronal perikarya, including the cytoplasm and plasma membrane of SGNs. The fluorescence intensity of NPR-A and NPR-C were more evident than that of our previous results, as we employed a different group of primary and secondary antibodies for labeling of ANP, NPR-A, and NPR-C. No specific immunoreactivity was seen in the negative controls for which the primary antibodies were omitted.

### ANP Promotes Neurite Outgrowth of SG Explants and Dissociated SGNs by Activating the NPR-A/cGMP/PKG Pathway

**FIGURE 3:**
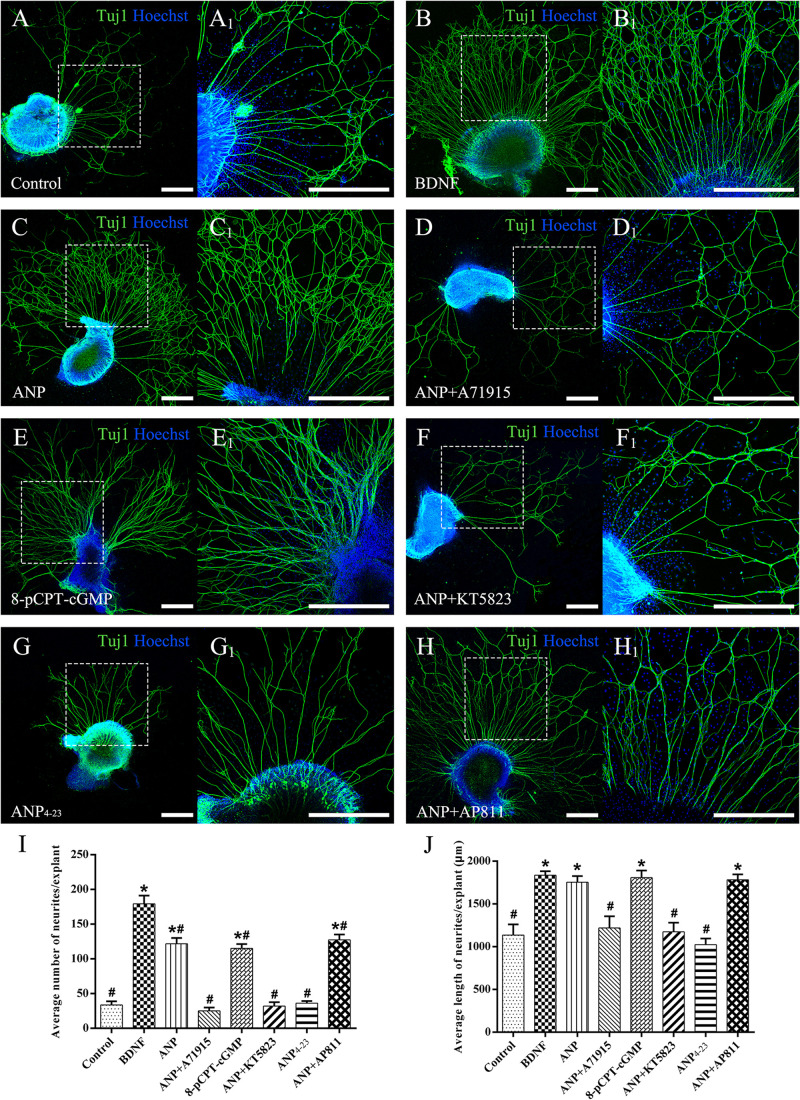
ANP promotes neurite outgrowth of SG explants by activating NPR-A/cGMP/PKG pathway. Cochlear SG explants were maintained in culture medium alone **(A)**, or medium supplemented with 20 ng/mL BDNF **(B)**, 1 μM ANP **(C)**, 1 μM ANP plus 1 μM A71915 **(D)**, 1 μM 8-pCPT-cGMP **(E)**, 1 μM ANP plus 1 μM KT5823 **(F)**, 1 μM ANP_4–23_ **(G)**, or 1 μM ANP plus 1 μM AP811 **(H)** for 7 days. Neurons were double-labeled with TuJ1 antibody (green) and Hoechst (blue). **(A_1_–H_1_)** High-magnification views of the boxed region of **(A–H)**. Scale bars = 500 μm. The number of neurites **(I)** and average neurite length **(J)** of explants were quantified (*N* = 5 explants for each group). Results are expressed as mean ± SEM (*P < 0.05, versus negative control samples; ^#^*P* < 0.05, versus positive control samples/BDNF).

To explore the possible effects of two distinct downstream signaling pathways of ANP receptors on the neurite outgrowth of SGNs, we initially counted the average number of regenerated neurites and the mean length of neurites extended from SG explants of P3 rats in each different distinct experimental group maintained in the culture medium containing specific reagents *in vitro* for 7 days (at DIV7). The explants treated with the culture medium alone were served as a negative control, while the explants that received the BDNF-containing medium for trophic support of neurite extension were used as a positive control. As shown in Figure 3, a small number of spontaneous neurite outgrowth with short length were observed in explants from negative controls (Figure 3A). As expected, robustly sprouting and elongating neurites were induced from explants treated with 20 ng/ml BDNF (Figure 3B). Interestingly, treatment of samples with 1 μM ANP elicited pronounced neurite outgrowth from SG explants (Figure 3C). For better understanding the possible mechanism of ANP in promoting the neurite outgrowth of SGNs, we discovered whether this polypeptide hormone acts via its GC-coupled receptor NPR-A or its clearance receptor NPR-C. In the presence of 1 μM NPR-A antagonist A71915, ANP (1 μM) failed to develop neurite elongation, suggesting that ANP-induced cGMP formation was abolished (Figure 3D), with respect to significantly less number and length of neurite than those in BDNF-positive control cultures. Treatment with 1 μM of the cell-permeable cGMP analog 8-pCPT-cGMP mimicked the effect of ANP-mediated neurite outgrowth, which would involve stimulation of PKG (Figure 3E). Conversely, application of 1 μM KT5823, a selective inhibitor of PKG, appeared to abrogate ANP-induced neurite outgrowth and sprouting (Figure 3F). Furthermore, incubation with 1 μM ANP_4–23_, a specific NPR-C agonist, did not significantly stimulate neurite outgrowth from SG tissues (Figure 3G). Despite blockade of NPR-C with its antagonist AP811, ANP considerably elevated the number and the length of elongating neurites (Figure 3H) in comparison to the negative controls.

**FIGURE 4:**
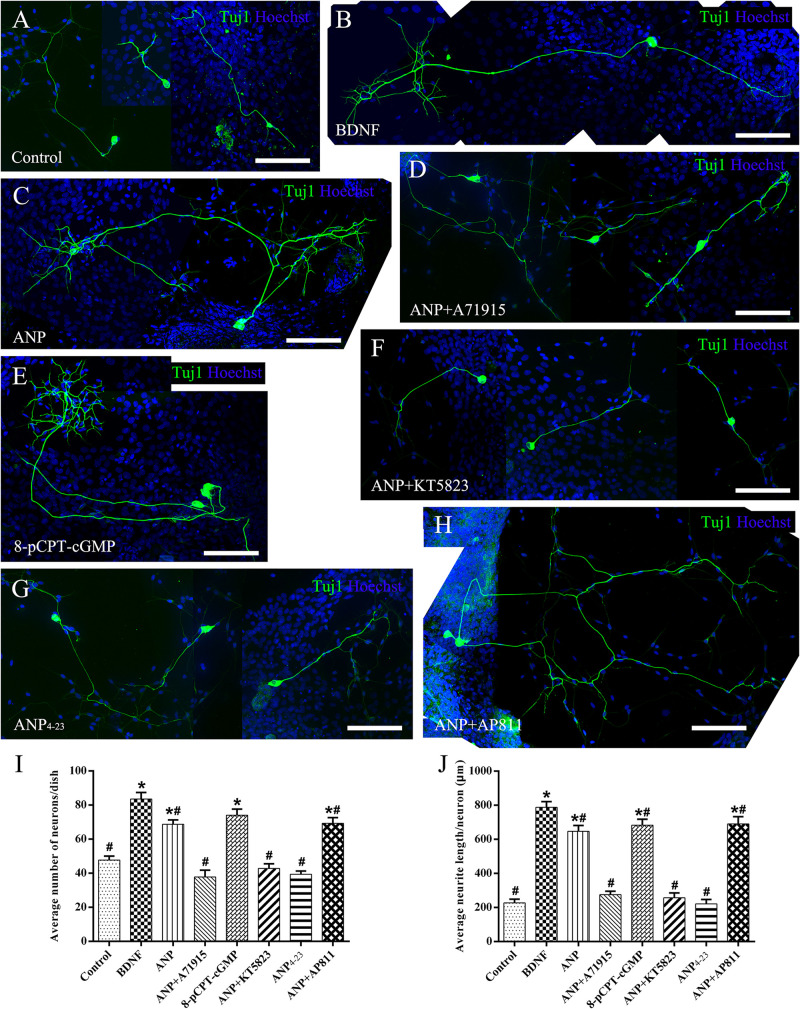
ANP promotes neurite outgrowth of dissociated SGNs by activating NPR-A/cGMP/PKG pathway. Dissociated SGNs were maintained in culture medium alone **(A)**, or medium supplemented with 20 ng/mL BDNF **(B)**, 1 μM ANP **(C)**, 1 μM ANP plus 1 μM A71915 **(D)**, 1 μM 8-pCPT-cGMP **(E)**, 1 μM ANP plus 1 μM KT5823 **(F)**, 1 μM ANP_4–23_ **(G)**, or 1 μM ANP plus 1 μM AP811 **(H)** for 5 days. Neurons were double-labeled with TuJ1 antibody (green) and Hoechst (blue). Scale bars = 100 μm. The cell number **(I)** and average neurite length **(J)** of dissociated SGNs were counted (*N* = 5 culture dishes for each group). Results are expressed as mean ± SEM (*P < 0.05, versus negative control samples; ^#^*P* < 0.05, versus positive control samples/BDNF).

Subsequently, we counted the mean neuronal number and the average neuritic process length of dissociated SGNs from P3 rats incubated in the culture medium with the assigned additives *in vitro* for 5 days (at DIV5). To provide a positive or negative control, the neuronal cultures were maintained in medium with or without the addition of BDNF, respectively. Representative images of neuronal cultures from various experimental groups are illustrated in Figure 4. The average number of neurons per culture dish was 47.7 ± 2.3, and the average neurite length per neuron was 227.2 ± 21.4 μm in negative control samples (Figure 4A). A significantly increased number of neurons and elongating neurite outgrowth were seen in cell cultures treated with 20 ng/ml BDNF (Figure 4B). As expected, the number of neurons and neurite length of ANP-treated (Figure 4C) and 8-pCPT-cGMP-treated (Figure 4E) neurons were significantly increased compared to the negative control samples. Conversely, 1 μM ANP failed to elevate the neuronal number and neurite elongation of SGNs in the presence of 1 μM A71915 (Figure 4D) or 1 μM KT5823 (Figure 4F). Furthermore, 1 μM ANP_4–23_ did not elicit the same effect as ANP (Figure 4G), while 1 μM AP811 did not suppress the effect of ANP on neuronal maintenance and neurite outgrowth (Figure 4H). Significantly, incubation with ANP (Figure 4C) and the cGMP analog 8-pCPT-cGMP (Figure 4E) inspired branch formation of neurites in cultured SGNs, and a similar phenomenon could be seen in BDNF-treated neurons (Figure 4B) and is previously described in our report (Sun et al., 2020), indicating the cascade in which neurotrophins and ANP/NPR-A/cGMP/PKG activation lead to a trophic support for neurite growth and branching. Accordingly, the mechanism of ANP/NPR-A/cGMP/PKG signaling in the regulation of axonal branching or pathfinding requires further exploration during cochlear development to comprehensively elucidate the principle underlying the assembly of auditory circuits.

Collectively, these observations indicate that ANP may promote outgrowth and regeneration of neurites from SGNs by activating the NPR-A/cGMP/PKG pathway. In addition, the variation tendency associated with the number of neurites from SG explants (Figure 3I) and cell number of dissociated SGNs (Figure 4I) suggested that the ANP/NPR-A/cGMP/PKG pathway may improve neuronal survival *in vitro*, to a certain extent.

### ANP Attracts Neurite Outgrowth of SG Explants by Activating the NPR-A/cGMP/PKG Pathway

**FIGURE 5:**
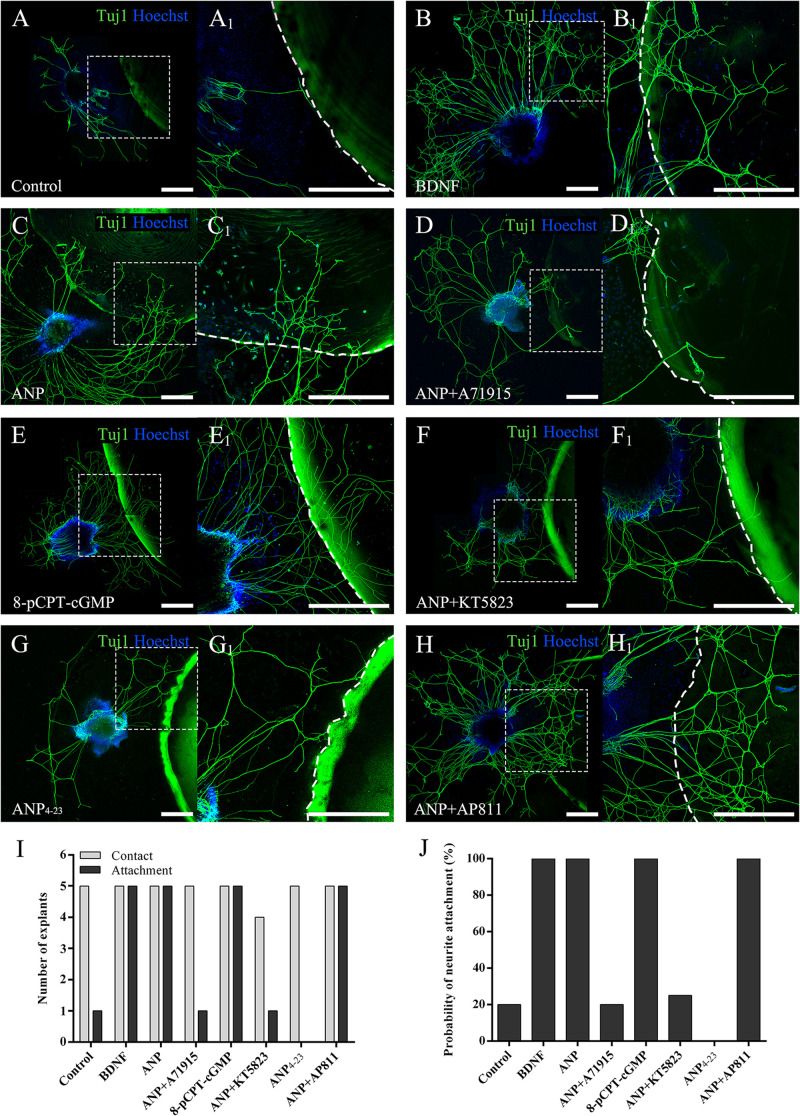
ANP attracts neurite outgrowth of SG explants by activating NPR-A/cGMP/PKG pathway. SG explants were settled in close proximity to droplets of 0.25% native PuraMatrix solution **(A)**, or 0.25% PuraMatrix mixture incorporated with 20 ng/mL BDNF **(B)**, 1 μM ANP **(C)**, 1 μM ANP plus 1 μM A71915 **(D)**, 1 μM 8-pCPT-cGMP **(E)**, 1 μM ANP plus 1 μM KT5823 **(F)**, 1 μM ANP_4–23_ **(G)**, or 1 μM ANP plus 1 μM AP811 **(H)** and maintained in culture medium for 4 d. Neurons were double-labeled with TuJ1 antibody (green) and Hoechst (blue). **(A_1_–H_1_)** High-magnification views of the boxed region of **(A–H)**. The dashed white line marks the border of the PuraMatrix droplet. Scale bars = 500 μm. The number of explants extended neurites to contact or attach to the droplet surface **(I)** and the probability of neurite attachment to PuraMatrix hydrogel **(J)** were determined (*N* = 5 explants for each group).

To evaluate the capacity to attract SGN neurites of downstream signaling pathways mediated by ANP and its receptors, we determined the probability of neurite attaching to surfaces of PuraMatrix hydrogel incorporated with given additives after SG explants of P3 rats were maintained *in vitro* for 4 days (at DIV4). As illustrated in Figure 5, a few neurites sprouting from explants contacted the edge of the PuraMatrix droplets but rarely grew onto the hydrogel surface (attachment) and thus yielded a low probability (20%) of neurite attachment to native PuraMatrix (Figure 5A). To attract extending neurites from explants, 20 ng/ml of BDNF was incorporated into 0.25% PuraMatrix, which gave rise to increased probability (100%) of neurite attachment (Figure 5B). Likewise, a substantial number of neurites were attracted and attached to surfaces of hydrogel incorporated with 1 μM ANP (Figure 5C). Similar patterns of neurite attachment could also be noticed on explants settled in proximity to hydrogel incorporated with 1 μM 8-pCPT-cGMP (Figure 5E). In contrast, incorporation of 1 μM NPR-A blocker A71915 (Figure 5D) or KT5823 (Figure 5F) into ANP-supplemented PuraMatrix resulted in drastically reduced probability of neurite attachment. Besides, activation of NPR-C with 1 μM ANP_4–23_ failed to support neurite attachment to hydrogel (Figure 5G), whereas blockade of NPR-C with 1 μM AP811 did not compromise the capacity of ANP-incorporated hydrogel to attract neurites (Figure 5H). These findings indicate a high capacity of ANP to attract neurites of SG explants by activating the NPR-A/cGMP/PKG pathway.

### ANP Promotes Neuronal Survival of Dissociated SGNs Against Glutamate Excitotoxicity by Activating the NPR-A/cGMP/PKG Pathway

**FIGURE 6:**
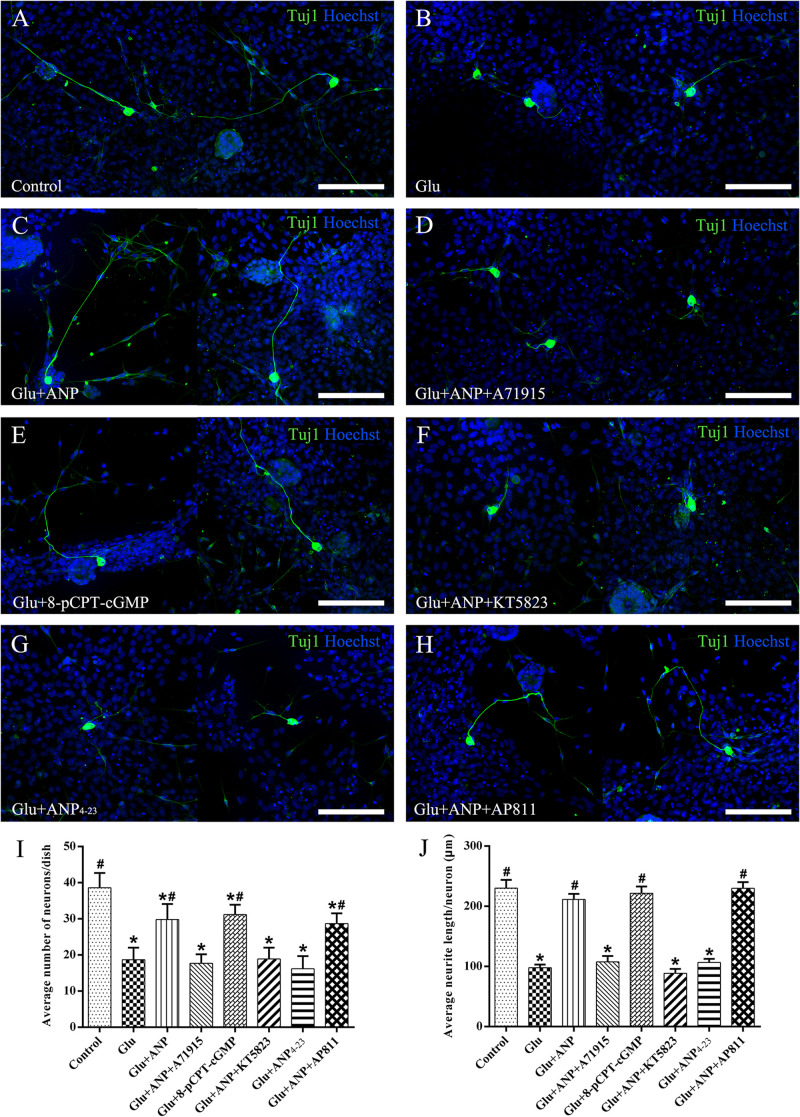
ANP promotes neuronal survival of dissociated SGNs against glutamate induced-excitotoxicity by activating NPR-A/cGMP/PKG pathway. Dissociated SGNs were incubated in culture medium alone **(A,B)**, or medium supplemented with 1 μM ANP **(C)**, 1 μM A71915 **(D)**, 1 μM 8-pCPT-cGMP **(E)**, 1 μM ANP plus 1 μM KT5823 **(F)**, 1 μM ANP_4–23_ **(G)**, or 1 μM ANP plus 1 μM AP811 **(H)** for 24 h. Then, 100 μM glutamate was added to the culture medium of all experimental cultures **(B–G)** and maintained for 48 h. Neurons were double-labeled with TuJ1 antibody (green) and Hoechst (blue). Glu: glutamate. Scale bars = 100 μm. The neuronal number **(I)** and average neurite length **(J)** of dissociated SGNs were calculated (*N* = 5 culture dishes for each group). Results are expressed as mean ± SEM (*P < 0.05, versus control samples; ^#^*P* < 0.05, versus glutamate treated samples/Glu).

To verify the possible neuroprotective effects of ANP receptor-associated downstream signaling pathways on SGNs against glutamate-induced excitotoxicity, we calculated the neuronal number and neurite length of dissociated SGNs from P3 rats cultured in medium containing the additives for 3 days (at DIV3). Representative images from cultures in different experimental groups are illustrated in Figure 6. The average number of neurons per dish was 38.6 ± 4.1, and the average neurite length per neuron was 234.0 ± 13.8 μm in control samples (Figure 6A). A drastic decrease in the number and neurite length of adhering neurons was observed in cultures subjected to excitotoxicity elicited by 100 μM glutamate for 48 h (Figure 6B), indicating that glutamate excitotoxicity would influence neuronal viability and neurite integrity, contributing directly to neurodegeneration (neuronal loss and neurite retraction) of SGNs. Pretreatment of dissociated SGNs with 1 μM ANP (Figure 6C) or 1 μM 8-pCPT-cGMP (Figure 6E) was able to substantially prevent neuronal loss and neurite retraction induced by glutamate exposure, whereas pre-incubation with 1 μM NPR-A inhibitor A71915 (Figure 6D) or 1 μM KT5823 (Figure 6F) abolished ANP-mediated neuronal survival and neurite outgrowth. Furthermore, 1 μM NPR-C agonist ANP_4–23_ failed to mimic the effect of ANP on neuronal viability and neurite integrity (Figure 6G), while 1 μM NPR-C blocker AP811 did not suppress the protective effect of ANP against glutamate excitotoxicity (Figure 6H). Accordingly, these results demonstrate that the neuroprotective effect of ANP on promoting survival of SGNs against glutamate-induced excitotoxicity is mediated by the NPR-A/cGMP/PKG pathway.

## Discussion

SGNs are auditory afferent neurons located in the Rosenthal canal in the cochlear modiolus, where they constitute the initial segment of auditory circuit to transmit neural impulse encoding acoustic information from the cochlea to the brain. The peripheral dendrites of SGNs extending from their somata within the modiolus form synaptic contacts with mechanosensory HCs in the organ of Corti, while their central axons connect to the cochlear nuclei within the brainstem (Nayagam et al., 2011). Many stress stimulators like ototoxic drugs and noise exposure had been testified to produce vast ROS in HCs and damage the HCs (Sun et al., 2014; Guan et al., 2016; Yu et al., 2017; Li H. et al., 2018; Zhang Y. et al., 2019; Gao et al., 2020; Zhong et al., 2020). Once the sensory epithelium of the cochlea or contacts with HCs are damaged due to auditory insults, SGNs may retract peripheral neurites and progressively degenerate due to lack of neurotrophic support (Stankovic et al., 2004; Vink et al., 2021). For individuals suffering from profound to severe SNHL, the treatment of choice at present is the implantation of a cochlear electrode array (CIs) by which the function of the missing HCs can be replaced. After insertion of the electrode into the cochlear scala tympani, auditory neurons can be stimulated electrically to create a hearing neural impulse. Benefits from the CIs are influenced by not only the amount of functional SGNs stimulated by the CIs but also the anatomical gap between auditory neurons and implanted cochlear electrodes. To establish an integrated neural-electrode interface, SGN neurites sprouting from cochlear bony structures must grow and be directed to the electrode surface. If neurites extend closer to the electrode and yield lower stimulus thresholds in a CI array, it might be possible to achieve controllable neural stimulation as well as improved sound quality and speech perception (Kwiatkowska et al., 2016; Li et al., 2017). To facilitate regeneration and guidance of peripheral processes from residual auditory neurons toward an implanted electrode, promising neurotrophic molecules (e.g., neurotrophins) are under research, due to their profound impact on neuronal survival, neurite outgrowth, and subsequent performance of CIs (Pfingst et al., 2017; Ma et al., 2019).

The cGMP signaling cascade has a versatile role in a broad spectrum of biological processes and maintains, in both the central and peripheral nervous systems, all aspects of neuronal functions including neurogenesis, synaptic formation, neuroprotection, neuronal excitability, and sensory transduction associated with olfaction, vision, and nociception (Zhao et al., 2009; Zhang et al., 2010). ANP and other members of the NP family, along with their receptors, are widely distributed in the mammalian nervous system and may participate in the regulation of neuroprotection, neural development, synaptic transmission, and information processing. Circumstantial evidence indicates the significance of cGMP signaling for neurite outgrowth and pathfinding and thus sheds light on the possible involvement of NPs in neuronal development and neurite regeneration. Particularly, cGMP signaling that is activated through binding of the ligand CNP to its transmembrane GC-coupled NPR-B (also known as NPR2 or GC-B) controls axonal bifurcation of cranial sensory ganglia neurons including cochlear SGNs entering the hindbrain (Lu et al., 2011, 2014; Ter-Avetisyan et al., 2014, 2018; Wolter et al., 2018; Schmidt and Fritzsch, 2019) and DRG neurons entering the spinal cord (Schmidt et al., 2002, 2007; Kishimoto et al., 2008; Schmidt et al., 2009; Zhao et al., 2009; Xia et al., 2013; Schmidt et al., 2016, 2018; Dumoulin et al., 2018a,b; Tröster et al., 2018). Likewise, knocking out PKGIα (also termed cGKIα) or NPR-B leads to defective central axonal projection of SGNs or DRG neurons in mouse models. Consequently, these studies suggested that the CNP/NPR-B/cGMP/PKG pathway might be implicated in the major events related to neurite outgrowth or pathfinding during neuronal development.

Additionally, increasing evidence indicates that NPs can exert neuroprotective functions by activation of GC-coupled NPRs. It has been reported that the ANP/NPR-A pathway might possess neuroprotective effects on rat retinal neurons against excitotoxicity caused by *N*-methyl-D-aspartate, probably by the activation of D1 dopamine receptors (Kuribayashi et al., 2006). CNP could serve a neuroprotective role which rescued rat retinal ganglion cells from apoptotic impairment *in vitro* or *in vivo* (Ma et al., 2010). ANP and BNP could inhibit apoptosis of cultured PC12 cells induced by serum deprivation and improve their survival with increasing cGMP levels (Fiscus et al., 2001). Pretreatment with ANP alleviated nitric oxide-induced neuronal apoptosis of a cholinergic-neuron-like cell line NG108-15, associated with elevation of cGMP levels (Cheng Chew et al., 2003). ANP could protect dopaminergic neuron-like models *in vitro* against neurotoxic insult for mimicking the neurodegeneration of Parkinson’s disease, which might be strongly dependent on the activation of the Wnt/β-catenin pathway (Colini Baldeschi et al., 2018). CNP was also shown to function as an innate neuroprotectant which improved neuronal survival of neonatal brain in mice from hypoxia–ischemia insult through its receptor NPR-B (Ma and Zhang, 2018).

Circumstantial evidence shows that ANP and receptors, i.e., its cognate receptor NPR-A and its clearance receptor NPR-C, are broadly distributed in mammalian CNS and peripheral sensory tissues, suggesting their involvement in regulation of various neuronal functions (Cao and Yang, 2008; Mahinrad et al., 2016). Taking together the results from previous findings and those of our works that demonstrate the colocalization of ANP and its receptors in cochlear SG, we proposed that ANP may also participate in the regulation of certain auditory neuronal functions through binding to its receptors (Lamprecht and Meyer zum Gottesberge, 1988; Furuta et al., 1995; Sun et al., 2013, 2014). Although our recent study suggests that ANP would stimulate neurite outgrowth of cochlear SGNs by triggering the NPR-A/cGMP/PKG pathway (Sun et al., 2020), the possible influence of NPR-C signaling within this process still needs to be examined, since NPR-C can interact with other second messenger signaling through the inhibition of adenylyl cyclase and activation of phospholipase C (Rose and Giles, 2008). In the present study, we employed organotypic explant and dissociated neuron cultures from SG of postnatal rats *in vitro* to evaluate the influence of ANP on neurite outgrowth, neurite attraction, and neuronal survival of SGNs. To determine which receptor is predominantly involved in the modulatory effects on neuronal functions of SGNs, we used the selective NPR-A antagonist A71915, cGMP analog 8-pCPT-cGMP, PKG inhibitor KT5823, selective NPR-C agonist ANP_4–23_, and NPR-C antagonist AP811. To explore the effect of ANP receptor pathways on neurite outgrowth of SGNs, we initially calculated the number and length of neurites extended from SG explants and dissociated neurons. Furthermore, the capacity of ANP receptor pathways to attract neurites was assessed by determining the probability of neurite attachment to the hydrogel surface by employing an *in vitro* SG explant model cultured next to PuraMatrix droplets (Frick et al., 2017). In these procedures, BDNF served as positive controls since it acts as a soluble neurotrophin to enhance neurite elongation, as well as a guidance cue to attract the growing neurites toward the hydrogel. The slow and sustained release of BDNF, cytokines, and small molecules from similar hydrogels suggests favorable release kinetics of the PuraMatrix hydrogel that might be utilized to establish a concentration gradient attracting attachment of neurites (Gelain et al., 2010; Frick et al., 2017). At last, the possible neuroprotective effect of ANP receptor pathways on SGNs was explored by calculating the neuron number and neurite length of dissociated SGNs exposed to glutamate excitotoxicity. As illustrated above, our data indicate that ANP would effectively stimulate neurite outgrowth and promote neuronal survival of SGNs by activating the NPR-A/cGMP/PKG cascade, since this activity can be mimicked by 8-pCPT-cGMP and abolished by KT-5823. Specifically, a cGMP signaling pathway composed of the ligand ANP, GC-coupled receptor NPR-A, and the downstream effector PKG could support and attract neurite outgrowth of SGNs. In addition, the variation tendency relevant to the number of neurites from explants and cell number of dissociated SGNs in samples analyzed for neurite outgrowth implied that the ANP/NPR-A/cGMP/PKG signaling cascade may improve neuronal survival. Furthermore, ANP might possess a high capacity to promote neuronal survival of SGNs against glutamate-induced excitotoxicity by activating the NPR-A/cGMP/PKG pathway.

Taken together, our study provides valuable information regarding the distribution of ANP and its receptors as well as their potential modulatory effects on neuronal functions of primary auditory neurons in the inner ear of postnatal rat. The neuroregenerative and neuroprotective nature of ANP/NPRA/cGMP/PKG-dependent signaling also strongly intimates that ANP could perform a vital role in normal neuritogenesis (sprouting, elongation, and branching) of SGN during development of the inner ear. Manipulation of cGMP levels and activation of PKG by activating ANP and receptors signals represent a potential therapeutic strategy to enhance regrowth of SGN neurites and support SGN survival, which promises to be a fruitful area for developing new and effective therapies for SNHL. Future investigations are necessary to unravel the details of this endogenous neuropeptide modulating the physiological functions of cochlear neurons, which will enhance our understanding of the mechanisms underlying normal and pathological states of hearing and provide future clinical applications on effective prophylactic and therapeutic treatment for hearing impairment.

## Data Availability Statement

The original contributions presented in the study are included in the article/supplementary material, further inquiries can be directed to the corresponding author/s.

## Ethics Statement

The animal study was reviewed and approved by the Animal Ethics Committee of the Fourth Military Medical University.

## Author Contributions

FS, KZ, and D-jZ conceived and designed the study. FS, K-yT, X-yZ, and WL performed the experiments. K-yT, JW, and C-pZ interpreted the data. FS and KZ wrote the manuscript. J-hQ and D-jZ reviewed and edited the manuscript. All authors have read and approved the final manuscript.

## Conflict of Interest

The authors declare that the research was conducted in the absence of any commercial or financial relationships that could be construed as a potential conflict of interest.

## Footnotes

1 https://www.who.int/activities/highlighting-priorities-for-ear-and-hearing-care
